# Supplementary material for: Digital assessment of walking ability: Validity and reliability of the automated figure-of-eight walk test in older adults
Source: PLoS One. 2025 Feb 10;20(2):e0316612. doi: 10.1371/journal.pone.0316612 (PMC11809801; doi:10.1371/journal.pone.0316612)
Supplement: Attachment — Submitted filename: Response to Reviewers.docx [file pone.0316612.s002.docx]

**Detailed Responses to Reviewer**

Dear Reviewer,

We really appreciate the reviewer’s detailed comments on our manuscript. The reviews have raised some excellent questions, and we sought to address each issue in detail in this letter and in the attached revised manuscript. You will find the reviewer’s comments in *italic*, followed by our detailed response. The original text is shown in blue, and the revised or added text is shown in red.

We hope that you will find our responses to be adequate. We thank you again for your time and effort spent reading and commenting on our manuscript.

Kind Regards,

Hyun-Ho Kong

Hyun-Chul Shon

December 06, 2024

1) *Introduction - what is the relevance of the 2nd paragraph, given that there is a test that measures curved path walk, the F8WT.*

: Thank you for your insightful comment regarding the relevance of the second paragraph in the Introduction. The purpose of this paragraph was to highlight the limitations of commonly used walking tests, such as short-distance walk tests and the Timed Up and Go test, which are primarily designed to assess straight-path walking or a single turn. These tests do not evaluate curved-path walking, which requires more complex motor skills, such as maintaining mediolateral balance and coordination, and is highly relevant to the activities of daily living in older adults.

By emphasizing these limitations, we aimed to introduce the Figure-of-Eight Walk Test (F8WT) as a superior clinical tool due to its ability to assess both straight-path and curved-path walking. This sets the stage for our study, demonstrating how F8WT bridges the gap left by other commonly used tests and makes it more clinically relevant. Furthermore, the second paragraph establishes the foundation for the study by highlighting the potential of automating the F8WT to enhance its utility and applicability, exceeding the capabilities of straight-path-focused evaluations.

While we understand your concern regarding the relevance of the second paragraph, we believe that this context is essential for establishing the rationale for our study. Therefore, we have decided to retain the original content without modification, as it directly supports the objectives of our research.

We hope this explanation clarifies the intent and relevance of the second paragraph in the Introduction. Thank you again for raising this important point.

2) *Introduction - the premise re the rationale for the study needs some more development; for example, several studies (e.g. https://pubmed.ncbi.nlm.nih.gov/30657232/, https://www.sciencedirect.com/science/article/abs/pii/S2211034822009348, https://linkinghub.elsevier.com/retrieve/pii/S0031940618301330) all have reported good inter-rater agreement, albeit in clinical populations. This appears to dent the claim re minimising inter-rater reliability (although the reasons re time-efficiency etc still hold true).*

: Thank you for your insightful comment. We agree with your observation that previous studies of manual measures of walking ability have demonstrated good inter-rater reliability for manual assessments, such as the F8WT, particularly in clinical populations. We consider that one of the primary reasons for the high inter-rater reliability reported in these studies is that the tests were conducted by trained technicians, such as physiotherapists, who are experienced in administering and evaluating these assessments.

The strength of using a digital device to automate physical function assessments lies in its ability to maintain high inter-rater reliability even when administered by individuals who are not trained technicians. The automated nature of the digital device eliminates subjective variability that may arise from human judgment and ensures consistent measurements regardless of the tester’s level of expertise. This feature is particularly valuable in non-clinical settings, such as community-based research or large-scale multicenter studies, where access to trained technicians may be limited.

Additionally, while inter-rater reliability is an important consideration, we emphasize that automation offers other significant advantages, including time efficiency, real-time data storage, and improved data traceability, all of which enhance the overall feasibility and scalability of using the F8WT in diverse research and clinical contexts.

We hope this explanation provides clarity and addresses your concerns. In response to your comment, we have revised the manuscript to reflect this point.

[Line 84-85]

Using a digital device to measure the F8WT can minimize inter-rater reliability issues commonly encountered in manual testing, making it a feasible option for multicenter cohort studies.

🡪

Using a digital device to measure the F8WT can minimize inter-rater reliability issues commonly encountered in manual testing, as it ensures high reliability even when administered by individuals who are not trained technicians.

3) *Please clarify that it is automated measure F8WT or manual measure F8WT throughout the manuscript.*

: Thank you for your valuable comment. We agree that it is essential to clearly differentiate between the automated (aF8WT) and manual (mF8WT) measurement methods to ensure the manuscript is easy to follow and avoids ambiguity. To address this concern, we carefully reviewed the manuscript and explicitly specified whether we are referring to the automated or manual F8WT in all relevant sections.

[Line 105-107]

or sensory deficits (visual or hearing impairment) that made it difficult to perform the F8WT or those who could not complete the test owing to personal reasons were excluded from the analysis.

🡪

or sensory deficits (visual or hearing impairment) that made it difficult to perform the aF8WT or those who could not complete the test owing to personal reasons were excluded from the analysis.

[Line 118-119]

The F8WT in this study was administered by a professional physiotherapist according to the protocol defined in the "National Fitness 100," a Senior Fitness Test (SFT) administered in Korea (26).

🡪

The mF8WT in this study was administered by a professional physiotherapist according to the protocol defined in the "National Fitness 100," a Senior Fitness Test (SFT) administered in Korea (26).

[Line 127-129]

The test was administered after one or two practice sessions, and the test was administered twice with an interval of at least 10 minutes, the earlier of which was defined as the result of the F8WT.

🡪

The test was administered after one or two practice sessions, and the test was administered twice with an interval of at least 10 minutes, the earlier of which was defined as the result of the mF8WT.

[Line 146]

The F8WT performed in this study was measured using the Care-Vision.

🡪

The aF8WT performed in this study was measured using the Care-Vision.

[Line 157-159]

Prior to the start of the F8WT, the DSFT system provided feedback by analyzing the skeletal position of the participant using a depth camera to ensure the correct starting position.

🡪

Prior to the start of the aF8WT, the DSFT system provided feedback by analyzing the skeletal position of the participant using a depth camera to ensure the correct starting position.

[Line 252-254]

Additionally, comparing the results of F8WT across studies is also challenging because there is currently no universally standardized protocol, leading to methodological differences between researchers.

🡪

Additionally, comparing the results of mF8WT across studies is also challenging because there is currently no universally standardized protocol, leading to methodological differences between researchers.

[Line 279-282]
Second, previous studies have used slightly different methods to conduct the F8WT (15-17), and this study was conducted using the method implemented in the Korean National Fitness 100 (26); therefore, the validity and test-retest reliability of the F8WT using other test protocol should be verified in the future.

🡪

Second, previous studies have used slightly different methods to conduct the mF8WT (15-17), and this study was conducted using the method implemented in the Korean National Fitness 100 (26); therefore, the validity and test-retest reliability of the aF8WT using other test protocol should be verified in the future.

4) *Statistical analysis - please clarify which tests are used for what for posterity.*

: Thank you for your insightful comment. We agree that clarifying the specific purpose of each statistical test will enhance the clarity and reproducibility of the manuscript. To address this, we have revised the Statistical Analysis section to explicitly state the purpose of each statistical test and its relevance to the study objectives. These revisions ensure that each statistical method is explicitly linked to its purpose within the study, making the rationale for their use clear and accessible for readers.

[Line 167-180]

The intraclass correlation coefficient (ICC) (2,1), a two-way random, single measure with absolute agreement, was evaluated to verify the degree of agreement between the results of the mF8WT by the assessor and the aF8WT using the DSFT system (27). Additionally, an ICC analysis was performed to verify the test-retest reliability of the aF8WT using the DSFT system (28). An ICC value of less than 0.40 was considered poor agreement, between 0.40 and 0.59 as fair, between 0.60 and 0.74 as good, and between 0.75 and 1.00 as excellent (29). In addition, Bland–Altman analysis was performed as another method to analyze the agreement between the mF8WT and aF8WT results, and the 95% confidence interval (CI) of the limits of agreement (LoA) was calculated (28). Pearson's correlation coefficient was also calculated to analyze the correlation between the results of the mF8WT and aF8WT, and the correlation coefficients were interpreted as negligible (<0.10), weak (0.10–0.39), moderate (0.40–0.69), strong (0.70–0.89), and very strong (0.90–1.00) correlations (30). The results of the mF8WT and aF8WT were graded according to age and sex as suggested by the Korean National Fitness 100, respectively, and the agreement of the participants' grades between the two methods was evaluated by calculating the Cohen's Kappa coefficient (31).

🡪

The intraclass correlation coefficient (ICC) (2,1), a two-way random, single measure with absolute agreement, was evaluated to verify the degree of agreement between the results of the mF8WT by the assessor and the aF8WT using the DSFT system, ensuring consistency between the two measurement methods (27). Additionally, an ICC analysis was performed to assess the test-retest reliability of the aF8WT, evaluating its consistency across repeated tests (28). An ICC value of less than 0.40 was considered poor agreement, between 0.40 and 0.59 as fair, between 0.60 and 0.74 as good, and between 0.75 and 1.00 as excellent (29). In addition, Bland–Altman analysis was performed to assess the bias and limits of agreement (LoA) between the mF8WT and aF8WT results, including the calculation of the 95% confidence interval (CI) for the LoA, to provide a detailed evaluation of measurement differences (28). Pearson's correlation coefficient was also calculated to determine the strength and direction of the linear relationship between the results of the mF8WT and aF8WT, providing additional insights into their correspondence. The correlation coefficients were interpreted as negligible (<0.10), weak (0.10–0.39), moderate (0.40–0.69), strong (0.70–0.89), and very strong (0.90–1.00) correlations (30). The results of the mF8WT and aF8WT were graded according to age and sex as suggested by the Korean National Fitness 100, respectively, and the agreement of the participants' grades between the two methods was evaluated by calculating the weighted Cohen’s Kappa coefficient. The interpretation of kappa values followed standard thresholds: less than 0.20 as poor agreement, 0.21 to 0.40 as fair agreement, 0.41 to 0.60 as moderate agreement, 0.61 to 0.80 as substantial agreement, and greater than 0.80 as almost perfect agreement (31).

5) *Cohen's kappa - was that weighted kappa (given there were more than two categories) and was there a threshold set for acceptable / good etc?*

: Thank you for your insightful comment. We appreciate the opportunity to clarify our use of Cohen’s kappa in this study. In our analysis, we used the weighted Cohen’s kappa to account for the ordinal nature of the F8WT grades. This method considers the degree of disagreement between categories, providing a more refined measure of agreement compared to the unweighted kappa. For interpretation, we applied standard thresholds for kappa values: less than 0.20 indicates poor agreement, 0.21 to 0.40 represents fair agreement, 0.41 to 0.60 indicates moderate agreement, 0.61 to 0.80 represents substantial agreement, and values greater than 0.80 indicate almost perfect agreement. These thresholds were used to classify the level of agreement between the physical function grades assigned by mF8WT and aF8WT. The classification is based on widely accepted guidelines for interpreting kappa statistics. To address your comment, we have revised the manuscript to explicitly state that weighted Cohen’s kappa was used and have included the thresholds for interpretation.

[Line 178-180]

The results of the mF8WT and aF8WT were graded according to age and sex as suggested by the Korean National Fitness 100, respectively, and the agreement of the participants' grades between the two methods was evaluated by calculating the Cohen's Kappa coefficient (31).

🡪

The results of the mF8WT and aF8WT were graded according to age and sex as suggested by the Korean National Fitness 100, respectively, and the agreement of the participants' grades between the two methods was evaluated by calculating the weighted Cohen’s Kappa coefficient. The interpretation of kappa values followed standard thresholds: less than 0.20 as poor agreement, 0.21 to 0.40 as fair agreement, 0.41 to 0.60 as moderate agreement, 0.61 to 0.80 as substantial agreement, and greater than 0.80 as almost perfect agreement (31).
