# Supplementary material for: Digital assessment of walking ability: Validity and reliability of the automated figure-of-eight walk test in older adults
Source: PLoS One. 2025 Feb 10;20(2):e0316612. doi: 10.1371/journal.pone.0316612 (PMC11809801; doi:10.1371/journal.pone.0316612)
Supplement: Attachment — Submitted filename: Response to Academic Editor.docx [file pone.0316612.s001.docx]

**Detailed Responses to Academic Editor**

Dear Academic Editor,

We really appreciate the detailed comments on our manuscript. The reviews have raised some excellent questions, and we sought to address each issue in detail in this letter and in the attached revised manuscript. You will find the reviewer’s comments in *italic*, followed by our detailed response. The original text is shown in blue, and the revised or added text is shown in red.

We hope that you will find our responses to be adequate. We thank you again for your time and effort spent reading and commenting on our manuscript.

Kind Regards,

Hyun-Ho Kong

November 15, 2024

1) *The manuscript is exploring the validity and reliability of the automated F8WT. The test itself is a functional, practical test that is performed with minimal equipment and therefore applicable in almost any environment. The proposed additions, however, make it costlier, more difficult to execute and without any apparent benefit. There is still a need for a supervisor (both for correct execution and safety of the participants), there is a need for additional equipment, with the main outcome still being time taken to complete the test and not additional information by the more sophisticated experimental set-up. Therefore, the conclusion of the test being of clinical significance and application is difficult to support.*

: We appreciate the very important comment of the reviewer. While there are multiple benefits to using our Digital Senior Fitness Test (DSFT) system for conducting the F8WT compared to manual measurement, we regret that these benefits were not clearly articulated in our initial submission, which may have led to some confusion. In the revised manuscript, we have provided a more detailed description of the specific advantages of performing the F8WT using the DSFT system.

Firstly, as the reviewer mentioned, the DSFT system for the F8WT does not require a supervisor to ensure correct execution (Manuscript Line 141). As described in Lines 139-148, the DSFT system enables participants to perform the test independently without assistance from an assessor. Once the participants position themselves in front of the digital device, pre-recorded visual and auditory instructions explaining the test protocol are automatically replayed as often as needed, allowing participants to understand the instructions fully. Additionally, before beginning the F8WT, the DSFT system provides feedback to ensure the participant assumes the correct starting position by analyzing their skeletal position through a depth camera. All 83 participants in this study were able to safely perform the aF8WT without a supervisor, with the exception of two participants who had a severe hearing impairment that made it difficult to hear the auditory instructions provided by the DSFT system (n=2) and two participants who abandoned the test due to unfamiliarity with testing using a digital device (personal reason, n=2).

Second, as the reviewer points out, using a digital device to perform physical function tests such as the F8WT requires additional equipment costs. However, we believe that using the DSFT system may be more beneficial when considering the benefit-cost, as the F8WT as well as several other tests related to physical function in older adults (grip strength, shoulder active range of motion, 2 minute step test, timed up and go test, short physical performance battery) can be assessed using a single digital device called the DSFT system without the need for trained technicians who require constant labour costs. In addition, because the DSFT system can automatically store and analyse the results of the tests on a cloud server, data from cohort studies, such as senior fitness tests conducted in each country (e.g. the Korean National Fitness 100 programme in Korea), can be managed efficiently and at a lower cost.

Lastly, because the DSFT system uses a depth camera for motion tracking, it can extract additional gait parameters, such as step counts, step length, and variability of step length during the F8WT. However, including an analysis of these gait parameters would significantly expand the scope of the study, and there are some cases of missing gait parameter data for certain participants. Therefore, we plan to report these findings in a subsequent paper. In clinical settings, walking ability in older adults is typically assessed by the time taken to perform the F8WT alone, without in-depth gait parameter analysis. Consequently, the lack of gait parameter analysis in this study does not restrict the primary aim of evaluating the DSFT system’s clinical applicability as set out in this research. We have revised the manuscript to address the reviewer’s comments.

[Line 82-84]

Despite our thorough literature search, no study has evaluated the validity and reliability of automated measurement of F8WT using a digital device yet.

🡪

Despite our thorough literature search, no study has evaluated the validity and reliability of automated measurement of F8WT using a digital device yet. Using a digital device to measure the F8WT can minimize inter-rater reliability issues commonly encountered in manual testing, making it a feasible option for multicenter cohort studies. In addition, a digital device enables automatic, real-time data storage and analysis, enhancing data traceability and integrity while reducing the need for manual data entry and error checking by researchers.

[Line 106-108]

A total of 92 participants were recruited, of whom 83 were finally included in the analysis, after excluding those who were unable to complete the test owing to underlying medical conditions (n=5), hearing impairment (n=2), or personal reasons (n=2).

🡪

A total of 92 participants were recruited, of whom 83 were finally included in the analysis after excluding those who were unable to complete the test due to underlying medical conditions (n=5), hearing impairment that made it difficult to hear the auditory instructions provided by the DSFT system (n=2), or unfamiliarity with testing using a digital device, leading them to abandon the test for personal reasons (n=2). All 83 participants included in the study were able to safely perform the aF8WT without a supervisor.

[Line 147-148]

The chair in which the participant sat was placed 2 m in front of the DSFT system, and the cone used as the return point was placed at a distance of 2.4 m on either side of the chair (Fig 1).

🡪

The chair in which the participant sat was placed 2 m in front of the DSFT system, and the cone used as the return point was placed at a distance of 2.4 m on either side of the chair (Fig 1). Prior to the start of the F8WT, the DSFT system provided feedback by analyzing the skeletal position of the participant using a depth camera to ensure the correct starting position.

[Line 261-264]

Second, previous studies have used slightly different methods to conduct the F8WT [15-17], and this study was conducted using the method implemented in the Korean National Fitness 100 [26]; therefore, the validity and reliability of the F8WT using other methods should be verified in the future.

🡪

Second, previous studies have used slightly different methods to conduct the F8WT [15-17], and this study was conducted using the method implemented in the Korean National Fitness 100 [26]; therefore, the validity and reliability of the F8WT using other methods should be verified in the future. Finally, although the DSFT system can measure gait parameters such as step count and step length, we did not include these in our analysis, as their inclusion would have significantly expanded the scope of this study. We plan to validate these additional gait parameters in a further study to assess the clinical applicability of the DSFT system.

2)

2-1) *It would be useful to offer a justification for the statistical analysis followed, both for the validity analysis (e.g. DOI: https://doi.org/10.1054/ptsp.1999.0001) and the reliability analysis (e.g. DOI: 10.2165/00007256-199826040-00002); for example, justification for the lack of bias assessment.*

: We appreciate the reviewer’s detailed comment. The rationale for selecting each statistical analysis used to verify the validity and reliability of the DSFT system in our study is described in the Methods section under Statistical Analysis (Lines 153 to 163). We have confirmed that our statistical approach aligns with the methods recommended in the review articles on validity and reliability analysis cited by the reviewer. Accordingly, in the revised manuscript, we have referenced these review articles related to our statistical methods. And we recognize the importance of bias assessment in detecting potential systematic errors in repeated measures. However, in this study, only two measurements were taken with a limited sample size, reducing the necessity and practicality of a formal bias assessment. Our primary aim was to establish agreement and reliability between the two methods rather than investigate potential systematic bias within subgroups. To minimize possible sources of bias, we implemented measures such as sufficient time intervals and practice trials to reduce learning effects or fatigue. Although a bias assessment was not explicitly conducted, we acknowledge its importance, particularly for future studies involving diverse cohorts, to detect any systematic differences between subgroups.

[Line 156-158]

The intraclass correlation coefficient (ICC) was evaluated to verify the degree of agreement between the results of the mF8WT by the assessor and the aF8WT using the DSFT system.

🡪

The intraclass correlation coefficient (ICC) _(2,1)_, a two-way random, single measure with absolute agreement, was evaluated to verify the degree of agreement between the results of the mF8WT by the assessor and the aF8WT using the DSFT system [27].

[Line 158-163]

Additionally, an ICC analysis was performed to verify the test-retest reliability of the aF8WT using the DSFT system. An ICC value of less than 0.40 was considered poor agreement, between 0.40 and 0.59 as fair, between 0.60 and 0.74 as good, and between 0.75 and 1.00 as excellent [27]. In addition, Bland–Altman analysis was performed as another method to analyze the agreement between the mF8WT and aF8WT results, and the 95% confidence interval (CI) of the limits of agreement (LoA) was calculated.

🡪

Additionally, an ICC analysis was performed to verify the test-retest reliability of the aF8WT using the DSFT system [28]. An ICC value of less than 0.40 was considered poor agreement, between 0.40 and 0.59 as fair, between 0.60 and 0.74 as good, and between 0.75 and 1.00 as excellent [29]. In addition, Bland–Altman analysis was performed as another method to analyze the agreement between the mF8WT and aF8WT results, and the 95% confidence interval (CI) of the limits of agreement (LoA) was calculated [28].

*2-2) Nonetheless, one aspect that must be discussed is the results in terms of the actual scores and the range they have produced. For example, how do the mean scores of ~30s compare to the literature (e.g. DOI: 10.1093/gerona/glaa035) and how sensitive / helpful a test can be with the wide 95%CI reported here?*

: We appreciate the reviewer’s detailed comment. As the reviewer pointed out, it is important to compare the mean scores and 95% confidence intervals (CI) of the F8WT from previous studies with those of our study. However, while the F8WT involves walking along both straight and curved paths, there is currently no standardized test protocol universally accepted among researchers. Consequently, the F8WT is conducted with slight variations in method across different researchers and countries. Direct comparisons between our study and previous studies with differing test protocols are therefore not feasible. Our study specifically followed the protocol used in the Korean National Fitness 100 (KNF100) program, which may differ slightly from those in prior studies. We selected the KNF100 program’s protocol because it provides large-scale normative data for older adults in Korea, segmented by age and sex. The primary outcome of our study was to compare the results of manual measurement (29.1±4.9 sec) using the KNF100 F8WT protocol with the automated measurement obtained by the DSFT system (29.8±4.9 sec). The 95% confidence intervals for the manual and automated measurements were 28.0 to 30.2 sec and 28.7 to 30.9 sec, respectively, showing minimal difference between the two. In the future, we plan to conduct validation studies using F8WT test protocols applied in other studies.

[Line 215-217]

In this study, the results of the aF8WT using the DSFT system showed excellent agreement with the results of the mF8WT traditionally performed by an assessor, and the error between the manual and automated measurements was within a clinically acceptable range.

🡪

In this study, the results of the aF8WT using the DSFT system showed excellent agreement with those of the mF8WT traditionally performed by an assessor, and the error between the manual and automated measurements was within a clinically acceptable range, indicating a level of agreement comparable to or exceeding that observed between manual measurements conducted by different technicians.

[Line 234-236]

Because no previous study to our knowledge has evaluated the F8WT using a digital device, the results of the aF8WT using the DSFT system cannot be directly compared with the results measured using other digital devices.

🡪

Because no previous study to our knowledge has evaluated the F8WT using a digital device, the results of the aF8WT using the DSFT system cannot be directly compared with the results measured using other digital devices. Additionally, comparing the results of F8WT across studies is also challenging because there is currently no universally standardized protocol, leading to methodological differences between researchers.

*2-3) Finally, there appears to be an attempt at assessing agreement between the classification of participants by the two systems, but no statistical analysis or discussion is presented. The purpose of the manuscript make these discussions vital before the claim for clinical relevance and application can be made in the Discussion.*

: We appreciate the reviewer’s comment regarding the lack of statistical analysis when comparing the F8WT classification by age and sex provided by KNF100 between the manual and automated measurements. In response, we have applied the Cohen’s Kappa method to assess the agreement in classification between the two measurement methods, and this analysis has been incorporated into the revised manuscript.

[Line 163-166]

Pearson's correlation coefficient was also calculated to analyze the correlation between the results of the mF8WT and aF8WT, and the correlation coefficients were interpreted as negligible (<0.10), weak (0.10–0.39), moderate (0.40–0.69), strong (0.70–0.89), and very strong (0.90–1.00) correlations [28].

🡪

Pearson's correlation coefficient was also calculated to analyze the correlation between the results of the mF8WT and aF8WT, and the correlation coefficients were interpreted as negligible (<0.10), weak (0.10–0.39), moderate (0.40–0.69), strong (0.70–0.89), and very strong (0.90–1.00) correlations [30]. The results of the mF8WT and aF8WT were graded according to age and sex as suggested by the Korean National Fitness 100, respectively, and the agreement of the participants' grades between the two methods was evaluated by calculating the Cohen's Kappa coefficient [31].

[Line 196-198]

Of all the participants, 71.1% (n=59) showed no difference in physical function grades between the mF8WT and aF8WT results.

🡪

Analysis of the agreement in grades between the mF8WT and aF8WT results yielded a Cohen’s kappa coefficient of 0.56, with 71.1% (n=59) of participants showing no difference in physical function grades between the two measurements.

3) *Ideally, specify the type of validity you are examining (or use the term 'method comparison'). Similarly, to avoid confusion, please use the relevant terms consistently (e.g. reliability vs agreement).*

: We appreciate the detailed comment. As the reviewer suggested, the revised manuscript clarifies the type of validity by using the term "method comparison." Additionally, we have revised relevant sections to ensure that the terms "reliability" and "agreement" are used consistently, minimizing potential confusion for the reader.

[Line 29-31]

This study aimed to verify the validity and reliability of the automated FW8T (aFW8T) using a digital device based on image analysis by comparing the results of the aF8WT with those of the manual F8WT (mF8WT).

🡪

The aim of this study was to verify the validity (method comparison) and test-retest reliability of the automated FW8T (aFW8T) using a digital device based on image analysis by comparing the results of the aF8WT with those of the manual F8WT (mF8WT).

[Line 86-88]

This study aimed to verify the validity and reliability of the F8WT using the DSFT system by comparing the results of automated F8WT (aF8WT) performed using the DSFT system with those of manual F8WT (mF8WT).

🡪

This study aimed to verify the validity (method comparison) and test-retest reliability of the F8WT using the DSFT system by comparing the results of automated F8WT (aF8WT) performed using the DSFT system with those of manual F8WT (mF8WT).

[Line 233-235]

However, a previous study of the inter-rater reliability of the technician-administered manual measurement of the F8WT in community-dwelling older adults reported an ICC of 0.90 (0.71–0.97) [15], which is similar to our results (ICC=0.95 [0.91–0.97]).

🡪

However, a previous study assessing the agreement between raters for the technician-administered manual measurement of the F8WT in community-dwelling older adults reported an ICC of 0.90 (0.71–0.97) [15], which is similar to our results (ICC=0.95 [0.91–0.97]).

[Line 258-261]

Second, previous studies have used slightly different methods to conduct the F8WT [15-17], and this study was conducted using the method implemented in the Korean National Fitness 100 [26]; therefore, the validity and reliability of the F8WT using other methods should be verified in the future.

🡪

Second, previous studies have used slightly different methods to conduct the F8WT [15-17], and this study was conducted using the method implemented in the Korean National Fitness 100 [26]; therefore, the validity and test-retest reliability of the F8WT using other test protocol should be verified in the future.
